# Fluorescent Nanodiamond Applications for Cellular Process Sensing and Cell Tracking

**DOI:** 10.3390/mi9050247

**Published:** 2018-05-18

**Authors:** Sandra Claveau, Jean-Rémi Bertrand, François Treussart

**Affiliations:** 1 Vectorology and Anticancer Therapies, UMR 8203, CNRS, Univ. Paris-Sud, Institut Gustave Roussy, Université Paris-Saclay, 94805 Villejuif, France; 2 Laboratoire Aimé Cotton, CNRS, Univ. Paris-Sud, ENS Paris-Saclay, Université Paris-Saclay, 91405 Orsay, France; jean-remi.bertrand@gustaveroussy.fr

**Keywords:** nanodiamond, nitrogen-vacancy defects, bioimaging, single particle tracking, biosensing

## Abstract

Diamond nanocrystals smaller than 100 nm (nanodiamonds) are now recognized to be highly biocompatible. They can be made fluorescent with perfect photostability by creating nitrogen-vacancy (NV) color centers in the diamond lattice. The resulting fluorescent nanodiamonds (FND) have been used since the late 2000s as fluorescent probes for short- or long-term analysis. FND can be used both at the subcellular scale and the single cell scale. Their limited sub-diffraction size allows them to track intracellular processes with high spatio-temporal resolution and high contrast from the surrounding environment. FND can also track the fate of therapeutic compounds or whole cells in the organs of an organism. This review presents examples of FND applications (1) for intra and intercellular molecular processes sensing, also introducing the different potential biosensing applications based on the optically detectable electron spin resonance of NV^−^ centers; and (2) for tracking, firstly, FND themselves to determine their biodistribution, and secondly, using FND as cell tracking probes for diagnosis or follow-up purposes in oncology and regenerative medicine.

## 1. Introduction

Nanodiamonds (NDs) are diamond phase carbon nanomaterials that were initially used for their strong abrasive properties and as lubricant additives [1] for industrial applications. They are produced on a large scale by different processes [2] that include explosion during the detonation stage [3], the milling of diamond microcrystals synthesized by high-pressure high-temperature process (HPHT), by plasma-assisted chemical vapor deposition (CVD) [4], or by laser ablation [5]. ND can be endowed with remarkable properties, in particular photoluminescence via embedded point defect with perfect photostability, yielding fluorescent NDs (FNDs) [6,7] with optically detectable magnetic resonance (ODMR) properties [8]. The fluorescence originates from nitrogen-vacancy defects that occur in neutral (NV^0^) or negative (NV^−^) charge states. ODMR can only be observed in NV^−^ defects. Furthermore, the NV defects within the ND lattice confer a very high resolution for imaging single molecules. NDs also benefit from having a large variety of surface functionalizations [9]. Pristine or functionalized ND have been repeatedly reported to be non-toxic for cells and organisms [10,11,12]. Considering this set of unique properties, NDs are considered as a valuable nanoparticle for biomedical applications to study intracellular processes and to follow the fate of molecule delivery when NDs are used as a drug delivery platform.

Epifluorescence confocal imaging is the simplest method used to observe fluorescent nanodiamonds due to an excitation at a wavelength of 532 nm and an emission between 600 and 800 nm for the NV defects. When in vivo or ex vivo actions are performed, time-delayed detection is then preferred. The radiative lifetime of NV centers in nanodiamonds of approximately 25 ns [13] is one order of magnitude longer than the one of tissue autofluorescence, which is about 2 ns. Therefore, by using sub-nanosecond pulsed laser excitation and time-resolved detection, rejecting most of the photon counting events originating from autofluorescence light is possible, thus significantly improving the signal over background ratio [14].

This short review focuses on some biomedical applications of fluorescent nanodiamonds, taking advantage of their unique photophysical properties to use them for (1) intracellular and molecular processes sensing and (2) for long-term tracking in organisms for therapeutic or diagnostic purposes.

## 2. Cellular Processes Sensing

### 2.1. Cell Internalization and Intracellular Trafficking

From their first application in cellular studies, NDs have been observed by various microscopy techniques to be internalized efficiently in a large variety of cellular types, from human cancer cell lines [15] to mouse hippocampal neurons [16]. Therefore, fluorescent NDs are effective for investigating various intracellular processes, by detecting their fluorescence and, in some cases, by detecting a colocalization with other biomarkers.

**Figure 1:**
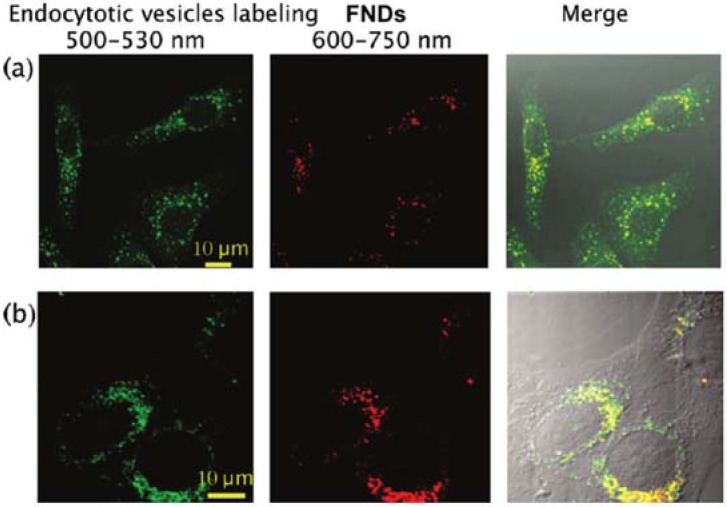
Localization of fluorescent nanodiamonds (FNDs) of size approximately 46 nm, in HeLa cells. Confocal fluorescence raster scan of HeLa cells incubated with FNDs in normal conditions then fixed. From left to right: raster scan in the green channel (500–530 nm) showing the endocytotic compartments, in the red channel (600–750 nm) showing the FNDs and a merged image of the two channels. (**a**) Colocalization study of FNDs with early endosomes labeled with EEA1-FITC (Fluorescein IsoThioCyanate) fluorescent conjugate, and (**b**) colocalization study of FNDs with lysosomes labeled with LysoTracker green dye. FNDs colocalized with endosomes or lysosomes appear in yellow in the merged image. Reprinted with permission [18]. Copyright (2009) American Chemical Society.

The first task involved identifying NDs’ internalization pathways. By using drugs that selectively inhibit the different uptake pathways, several studies highlighted that clathrin-mediated endocytosis was the dominant internalization process of HPHT nanodiamonds smaller than 100 nm [17,18]. The NDs were also revealed to only be located in the perinuclear region of the cytoplasm, and not in the nucleus [18], as presented in Figure 1. Furthermore, the smallest, isolated particles were observed to be free in the cytoplasm, when the bigger particles and aggregates were localized in the intracellular endocytic vesicles. Besides, the shape of a HPHT diamond nanoparticle appears to affect its internalization, trafficking inside the cell, and excretion from it [19,20]. Nevertheless, such behaviors are still debated in the scientific community.

In addition, a comparison of the internalization process between positively charged amine functionalized FND (FND-NH_2_) and transferrin grafted FNDs (FND-Tf) showed that the latter, which triggers receptor-mediated endocytosis, has a higher uptake efficiency compared to the former, which probably acts via electrostatic interactions between the NDs and the cell membrane [21]. This study highlighted the fact that the surface chemistry of nanodiamonds influences the internalization process and possibly the intracellular location. This was further documented by Alhaddad et al. [17] who showed that different cationic polymer surface coatings, such as Poly-EthyleneImine (PEI) vs. Poly-Allyleamine Hydrochloride (PAH), used to load siRNA on NDs by electrostatic interaction led to different internalization pathways. PAH coating involved clathrin-mediated endocytosis only, whereas PEI-coating experienced both clathrin-mediated endocytosis and micropinocytosis. The latter process allowed the siRNA-ND complexes to be engulfed in macropinosomes that have some leakage allowing the release of siRNA into the cytoplasm where it is active. The fluorescence of nanodiamonds served to precisely localize the FND in the cells by confocal microscopy, and to estimate the release dynamics of fluorescently-labeled siRNA.

To direct drug transport by FND to the deleterious cells with better efficacy, targeting moieties were grafted to the nanodiamond surface. Transferrin (Tf) is an ideal candidate molecule for such targeting as it has receptors on a large variety of cancer cells. Taking advantage of FND fluorescence, Li et al. demonstrated that the grafting of Tf at the surface of FND improved their internalization in HeLa cells [22]. They also reported that when cell’s Tf receptors were pre-saturated by free Tf, FND uptake decreased, indicating that it is a Tf-receptor-mediated process. This targeting strategy was then applied to vectorize Doxorubucin (Dox), a classical anti-cancer drug, with Tf-FNDs, which demonstrated the high specificity to cancer cells compared with normal cells, due to the active targeting [23]. Indeed, free Dox was more toxic (reduction of cell viability) on normal cells when it is free that when vectorized by FND-Tf, but FND-Tf-Dox was as toxic as free Dox on cancer cells.

### 2.2. Sensing Intra- and Inter-Cellular Biological Processes

In addition to the observation of their internalization in cells, the perfectly stable fluorescence conferred by the NV centers to NDs is particularly well suited for tracking intracellular processes such as endosomal transports. This process is impeded in neurodegenerative diseases, such as Parkinson and Alzheimer diseases [23]. The latter is detected at a too-advanced stage to be efficiently treated, so a need exists for the development of new unbiased approaches for earlier diagnosis. FND was used for this purpose by Haziza et al. [16]. The authors measured the intraneuronal transport parameters by tracking FNDs internalized in endocytic vesicles that were moved along the microtubule cytoskeleton by molecular motors. They demonstrated differences in endosomal transport parameters, such as velocity and motor stopping duration and frequency, between control neurons and neurons from transgenic mouse lines, mimicking a genetic risk factor for brain disease [16]. This study showed that the perfect photostability, the high brightness, and very low toxicity of FNDs allow the sensing of the impact of a very small protein concentration change in relation to neurodegenerative diseases.

Intercellular communication is essential in various biological processes, such as immunity, tissue regeneration, and development. This communication is completed through different gateways, including chemical synapses, gap junctions, and tunneling nanotubes (TNTs) [24]. The latter was investigated to be used as a delivery channel between various cells, being from identical or different types. A study was conducted on the communication between human embryonic kidney cells themselves or with human neuroblastoma cells, made through TNTs [25]. FNDs were coated with Bovine Serum Albumin (BSA) or with a green fluorescent protein (GFP) to track the colocalization and transport dynamics of the FNDs with the protein and their transport dynamics. The protein-loaded FND entered cells, first moved randomly inside the cytoplasm and then, after entering the TNT, continued with a directed motion, indicating a molecular-motor-mediated process. Furthermore, stop-and-go and to-and-from motions were observed under 100 µm, for both homo- and heterotypical intercellular transport, paving the way for a new delivery route in cancer therapy.

### 2.3. Labelling Cell Membrane in Culture and Probing Receptor Dynamics

Acid-cleaned FNDs have been reported to be spontaneously internalized, but in some cases, only the labeling of the cell membrane is desired without the internalization of the nanodiamonds. Such labeling is made with PEG-modified FNDs that are covalently coated with neoglycoproteins or with streptavidin. One application of this function is immunostaining to label the membrane of specific cells, including hepatocytes, which can be further sorted by flow cytometry [26]. 

Such functionalization and labeling was also used to study some transmembrane signaling functions and membrane endogenous proteins dynamics in live cultured-cells. Sotoma et al. [27] targeted the Interleukin-18 receptor alpha (IR-18Rα) chains on the plasma membrane of human embryonic kidney cells, and demonstrated that recording receptor trajectories at the cell surface is possible by grafting a beta-lactase-tag system on FNDs, specifically targeting IL-18Rα. In a more advanced experiment, Liu et al. investigated the trafficking dynamics and cell signaling of TGF-beta receptor [28]. The former is critical for the development of cancer targeting therapies. They functionalized FNDs with NH_2_-PEG-COOH, on which streptavidin and then BSA and biotin-TGF were attached. This complex efficiently labeled TGF-beta receptors, and the authors were able to track their three-dimensional (3D) motion with a high localization accuracy (10 nm in *x* and *y* and 19 nm in *z*). The trajectory analysis highlighted three different behavioral states of TGF-beta receptors: immobile, intermediate, and fast diffusion [28]. They showed that specific treatment with small molecule kinase inhibitors could change the dynamics of TGF-beta from immobile to intermediate and fast diffusion states, providing a better comprehension of the mechanisms of the TGF-beta pathway and helping the development of new therapeutics targeting TGF-beta-associated cancers.

### 2.4. Prospects in Biosensing Based on Electronic and Quantum Properties of NV^−^ Center in Nanodiamonds

The NV center charge state in a nanodiamond depends on the Fermi level of the host matrix, which can be adjusted by varying the surface functionalization. The oxygen-terminated FND favors emission of negatively charged NV^−^, whereas the hydrogen-terminated surface only emits neutral NV^0^ [29]. Each charge state population is quantified by the strength of its specific narrow zero-phonon line in the photoluminescence (PL) spectrum. The change in proportion of NV^−^ to NV^0^ PL intensity has been used in a purely optical method to detect the release in the cytoplasm of negatively charged nucleic acid transported by cationic polymer-coated FNDs [30].

NV centers in diamonds are remarkable not only for their perfectly stable fluorescence but also for the optically detectable electron spin resonance of the negatively charged form. In the presence of an external magnetic field, the NV^–^ electron spin resonance is shifted by the Zeeman effect, providing an optical means for magnetometry [31], or for orientation tracking of FNDs, even in the cellular environment [32]. NV-magnetometry with a bulk diamond substrate was successfully used to detect action potentials in the giant axon of a marine worm [33]. Although very promising, this approach cannot be easily extended to nanodiamonds whose matrix contains more impurities and defects than bulk diamond, leading to broader spin resonance and smaller spin coherence duration. However, another strategy relying on spin-to-charge conversion could enable action potential sensing with nanodiamonds placed very close to the source of the magnetic field [34]. The NV^−^ center electron spin resonance frequency also experiences a shift upon a thermal variation that allows the measurement of temperature changes with a noise as small as 9–10 mK·Hz^−1/2^ [35,36], in a range of temperatures from 5.6 K to 295 K [37]. The localization precision of the measurement is potentially in the range of a few tens of nanometers [38], making FND well suited for sensing temperature fluctuations at the subcellular resolution in living cells [35,39].

Furthermore, the state of the NV^–^ electron spin can be coherently manipulated with light and microwave at the ODMR microwave frequency, which provide additional sensing approaches. In particular, modification of the spin longitudinal relaxation time *T*_1_ can be measured by purely optical manipulations [40,41,42]. *T*_1_ reflects the magnetic noise surrounding the electron: an increase in noise results in a decrease in *T*_1_. Taking advantage of these properties, Rendler et al. [43] performed pH and redox potential sensing in conditions mimicking the intracellular environment. To achieve this goal, they attached paramagnetic gadolinium complexes (a high-spin source of strong magnetic noise) to FNDs by pH-dependent hydrolytically cleavable or reductively cleavable linkers. The change in pH or redox potential was then translated into a reduction of the relaxation time. This result creates the possibility of imaging physiological processes by purely optical means with high localization precision. In addition, the NV^−^ center in diamond spin decoherence can also be used to improve the sensitivity of electromagnetic field detection, which has been proposed for the real-time monitoring of membrane ion channel activity [44]. McGuinness et al. [45] completed a proof of concept experiment in which the coherence relaxation time *T*_2_ of NV^−^ spins in 45-nm FNDs was monitored with spin echoes sequences during the addition of Mn^2+^ ions in solution. The authors measured a decrease in *T*_2_ induced by only about 2500 Mn^2+^ ions surrounding the FND. This type of approach was further improved, leading to the detection of only 12 ferritin molecules at the FND surface [46,47] using *T*_1_ relaxometry and *T*_2_ measurements. The challenge of translating these first experiments into real biosensing applications relies on making them compatible with the complex ionic environment of the biological medium.

## 3. Long-Term Tracking of Biological Processes

### 3.1. Tracking the Fate of Molecules and Their ND Vector in Organisms

Nanodiamonds have been studied since the late 2000s as biomolecule delivery agents due to their high biocompatibility [12] and versatile surface functionalization [48]. The surface chemical groups can be modified and functionalized, modulating the surface electronic charges. Cationic or anionic charges can be created by physico-chemical methods [49,50], by wet chemical treatments [51], or by covalently grafting different functional groups [9]. The various functionalizations allow the fixing and delivering in various living model organism cargos, including drugs (doxorubicin [52], paclitaxel [53], water-insoluble compounds [51], etc.), nucleic acids and oligonucleotides [54,55,56], or DNA [57,58]. For instance, Doxorubicin can be physisorbed at the surface of functionalized NDs, allowing a slow and sustained release [52], enhancing tumor size reduction and mouse survival when compared with Doxorubicin injected with no vector.

When using ND for drug delivery in model living organisms like mice [52] (preclinical evaluations), a good understanding of their tissue distribution and elimination is required given their potential application in humans. This could be achieved due to the radioisotope labeling of nanodiamonds (^188^Re-DND [59], ^18^F-DND [60], ^125^I-DND [61], and ^99^Tc-DND [62]) that provides a qualitative distribution in the different organs. FNDs have been shown to preferentially accumulate in the lungs, liver, and spleen. This method is technically demanding and can only provide semi-quantitative information on the biodistribution. A more quantitative distribution was developed by Su et al. [63] by using the fluorescence of the NV centers and by observing them in organ sections with a time-gating microscopy imaging system. A qualitative insight in FND biodistribution was also obtained by Vaijayanthimala et al. [64] using an in vivo imaging system. After only a few minutes, FNDs spreading around the injection site could be monitored. The fluorescence intensity in different organs was then measured ex vivo with the same apparatus. The use of intrinsic fluorescence of FND for tissue distribution measurement appears to be a viable alternative to radiolabeling.

**Figure 2:**
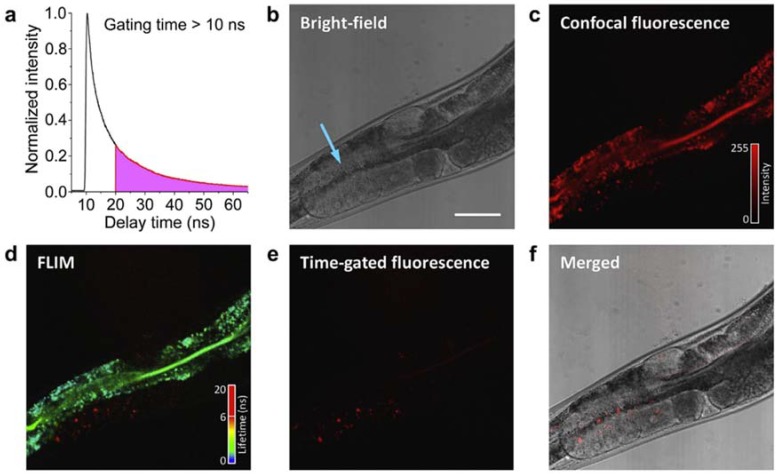
Observation of green fluorescent protein (GFP):Yolk lipoprotein complexes (YLC)-fluorescent nanodiamonds (FNDs) in *Caenorhabditis elegans* by fluorescence lifetime imaging microscopy (FLIM). (**a**) A fluorescence decay time trace of 100-nm FNDs excited by a picosecond pulsed laser; (**b**) bright field image, blue arrow indicates the site of injection; (**c**) confocal fluorescence image; (**d**) FLIM image; (**e**) time-gated fluorescence image at a delay greater than 10 ns; and (**f**) merged image of (**b**,**e**). Scale bar: 50 µm. Reprinted with permission [65]. Copyright (2012) Elsevier Ltd.

FNDs were also used to track the fate of proteins within an organism. Kuo et al. [65] followed the equivalent of low-density lipoproteins (LDLs) and Yolk lipoprotein complexes (YLC) in *Caenorhabditis elegans* worm during 12 h due to their coupling to FND. Time-delayed fluorescence imaging was used to achieve a higher signal over background imaging of FND in *C. elegans* compared with confocal fluorescence (Figure 2). This technique, combined with the perfect photostability of FNDs, allowed the tracking of the accumulation of YLC-FND in the multi-cellular embryos derived from the oocytes after a 12 h incubation period [65].

Nitrogen-vacancy defects have also been used to conduct long-term analysis, such as tracking of cell division and stem cell differentiation, or for diagnosis and prognostic purposes in oncology, like metastasis tracking.

### 3.2. Regenerative Medicine and Cancer Diagnosis Applications

Several pathologies that cannot be treated by pharmaceutical molecules could benefit from stem cell therapies [66,67]. This personalized medicine is based on the ability to restore a function by artificially differentiating stem cells into functional cells of choice. This therapeutic axis is mainly used for neurological disorders, such as Parkinson’s disease [68], or to support or sustain failing organs [69]. The use of such therapies has been welcomed with enthusiasm but determining biodistribution to characterize the basic features is still required. Assessing such distribution used to be complicated, particularly in vivo. However, owing to nanotechnology, this hurdle is being minimized. FNDs can be used to label whole cells by taking advantage of the efficient cellular uptake in most cell lines, their low exocytosis [70] (ensuring a large signal even after few divisions), and their very low toxicity, including toward stem cells [71]. FNDs provide a suitable platform to track stem cell fate, differentiation state and rate, and to assess the distribution of different cells in the tissues, as was performed in the context of lung regeneration [72].

**Figure 3:**
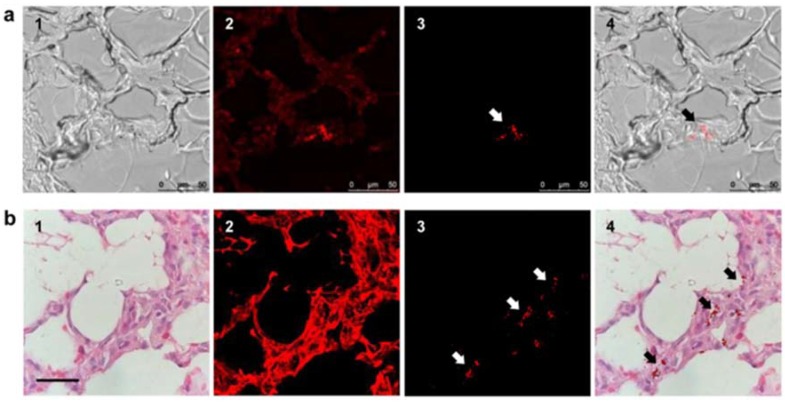
Fluorescence imaging of FND-labeled mesenchymal stem cells (MSC) in pig tissues. (**a**) Fast screening of a lung tissue before deparaffinization to find FND-labeled MSCs: (**1**) bright-field image, (**2**) fluorescence image without time-gating, (**3**) fluorescence image with time-gating at delays greater than eight nanoseconds, and (**4**) merged images of (**1**,**3**). Scale bar: 50 µm. (**b**) Identification of FND-labeled MSCs in a lung tissue section by confocal microscopy: (**1**) Hematoxylin & Eosin staining image, (**2**) confocal fluorescence image without time gating, (**3**) time-gated confocal fluorescence image, and (**4**) merged image of (**1**,**3**). Scale bar: 20 µm. Reprinted with permission [63]. Copyright (2017) Springer Nature.

Tracking of human placenta choriodecidual membrane-derived mesenchymal stem cells (pcMSCs) was completed in small pigs [63]. This study used two methods to detect and measure the FND amount in different organs to eventually measure the amount of pcMSCs. To determine the quantitative biodistribution of FND-labeled pcMSCs, the collected organs were first chemically digested and then the fluorescence of the FND was measured. By using a magnetically modulated fluorescence acquisition that uses the magneto-optical properties of NV^−^ centers, they managed to isolate the FND fluorescence signal from the background noise. With this method, they observed that the stem cells preferentially accumulated in the lungs, and then to a lesser extent, in the liver [63]. Furthermore, they managed to observe, with single-cell resolution, the FND-labeled pcMSCs cells in lung tissue sections, as shown in Figure 3, allowing the precise determination of the fate of pcMSCs after 24 or 48 h incubation in small pigs.

**Figure 4:**
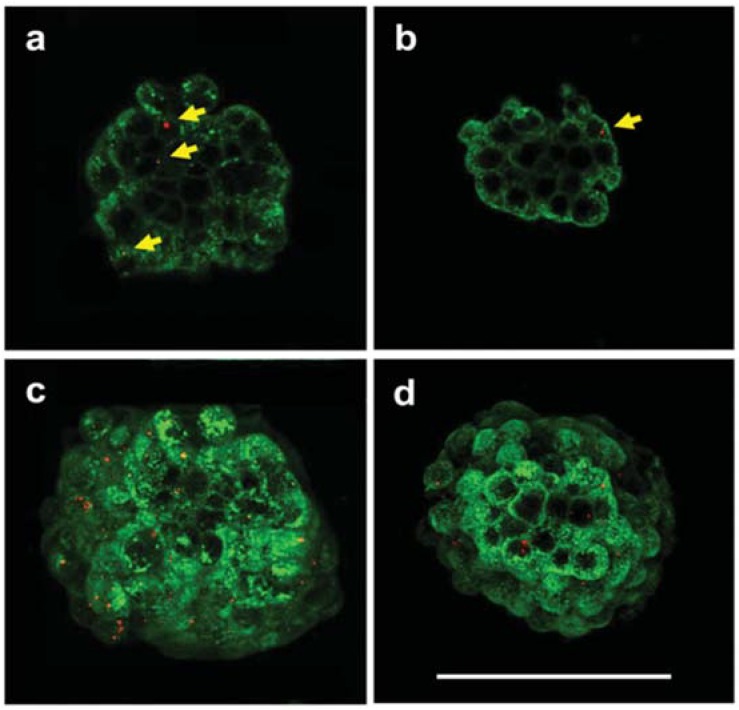
Confocal fluorescence imaging of two stages of development of quiescent cancer stem cell mammospheres labeled with FNDs. (**a**,**c**) primary stage and (**b**,**c**) secondary stage. Both mammospheres were stained with a cell membrane marker, Fluorescein IsoThioCyanate (FITC)-conjugated wheat germ agglutinin (green), and FNDs appear as the sparse red dots in cells. (**a**,**b**) single-section images, and (**c**,**d**) maximum projection images of a stack. Yellow arrows indicate the internalized FNDs. Scale bar: 100 µm. Reprinted with permission [73]. Copyright (2015) Wiley-VCH.

FNDs were also used to label and then track quiescent cancer stem cells (CSCs) [73]. CSCs are suspected to increase drug resistance in certain cases and are known to be responsible for tumor initiation, growth, and recurrence [74]. In addition, some CSCs are maintained in a quiescent state, preserving their self-renewal capacity after treatment [75]. The study of these cells using dye-labelled biomarkers is complicated and does not allow tracking in organs over a long time period. Lin et al. [73] used a model of CSCs labelled by endocytosis of FNDs and showed that such labeling outperforms conventional biochemical methods in regards to long-term tracking capability. Using a mammosphere assay, the authors observed that, even after 20 days, FND could still be detected in a subpopulation of cells, corresponding to slow-proliferating/quiescent cancer stem cells [73] (Figure 4). Furthermore, the sentinel lymph nodes (SLN), playing a key role in monitoring cancer progression, were also labelled with FND [64]. Indeed, the first metastases are suspected to develop in SLN. Their biopsy should then provide prognosis data. In this case, FND labelling of SNL should facilitate the biopsy. 

After assistance in diagnosis, support in the follow-up of the proliferation of the cancer is necessary. To this aim, Hui et al. [76] imaged individual FND-labeled cells circulating in whole blood by using a microfluidic circuit combined with fluorescence microscopy. Moreover, by applying the time-delayed detection modality (Figure 2a), these cells were still observed through a thin layer of chicken breast skin mimicking in vivo imaging conditions in an autofluorescent medium.

Together, these studies are improving accurate tumor cell and metastasis detection. We can envision the development of diagnostic devices based on tracking circulating tumor cells (CTC) in patients’ blood after labeling of the tumor with fluorescent nanodiamonds.

## 4. Conclusions

Nanodiamonds containing NV centers appear to be valuable bioimaging and biosensing probes. They allow the observation, characterization, and measurement of subcellular processes, from cellular uptake to intracellular physico-chemical parameters. NDs are also valuable for biomolecular dynamics sensing. Fluorescent nanodiamonds have also shown superior properties for the long-term tracking of stem cells or cancerous cells, offering possible applications in disease status monitoring. Despite the promising biomedical results presented in this review, a bottleneck still exists for fully in vivo live observation and imaging of fluorescent nanodiamonds. In vivo observations are complicated by the high autofluorescent background from tissues. This could be circumvented by taking advantage of the specific ODMR response of NV^−^ centers that allow the selective imaging of NV-containing nanodiamonds in an autofluorescent background [77,78]. A simpler version of this approach—although less sensitive—that only requires magnetic field modulation [79] was successfully used to detect FND in tissue with high contrast with wide field microscopy in vivo. When considered altogether, the remarkable physico-chemical and photophysical properties of fluorescent nanodiamonds makes them a unique and highly versatile nanoprobe for bioimaging and biomedicine.
